# Interpretable optimisation-based approach for hyper-box classification

**DOI:** 10.1007/s10994-024-06643-7

**Published:** 2025-02-06

**Authors:** Georgios I. Liapis, Sophia Tsoka, Lazaros G. Papageorgiou

**Affiliations:** 1 The Sargent Centre for Process Systems Engineering, Department of Chemical Engineering, UCL (University College London), Torrington Place, London, WC1E 7JE UK; 2 Department of Informatics, King’s College London, Bush House, London, WC2B 4BG UK

**Keywords:** Mathematical programming, Data classification, Mixed integer optimisation, Hyper-box, Interpretable machine learning

## Abstract

Data classification is considered a fundamental research subject within the machine learning community. Researchers seek the improvement of machine learning algorithms in not only accuracy, but also interpretability. Interpretable algorithms allow humans to easily understand the decisions that a machine learning model makes, which is challenging for black box models. Mathematical programming-based classification algorithms have attracted considerable attention due to their ability to effectively compete with leading-edge algorithms in terms of both accuracy and interpretability. Meanwhile, the training of a hyper-box classifier can be mathematically formulated as a Mixed Integer Linear Programming (MILP) model and the predictions combine accuracy and interpretability. In this work, an optimisation-based approach is proposed for multi-class data classification using a hyper-box representation, thus facilitating the extraction of compact IF-THEN rules. The key novelty of our approach lies in the minimisation of the number and length of the generated rules for enhanced interpretability. Through a number of real-world datasets, it is demonstrated that the algorithm exhibits favorable performance when compared to well-known alternatives in terms of prediction accuracy and rule set simplicity.

## Introduction

Data mining refers to the analysis of data so as to extract patterns and predict outcomes. One of the prominent subjects of data mining is classification, where given a number of samples that are characterised by certain independent variables and their class membership, the objective is to identify the correlations and assign a new sample to a class based on its attributes. Data classification models have been applied to a wide range of research areas, including but not limited to healthcare (Ainali et al., 2012; Yang et al., 2014; Letham et al., 2015; Ustun & Rudin, 2016; Bertsimas et al., 2016; Souri & Tsoka, 2020; Sevinç 2022; Souri et al., 2023), fault diagnosis and predictive maintenance (Li et al., 2014; Wang & Ma, 2014; Jung et al., 2018; Carvalho et al., 2019; Ouadah et al., 2022) and financial analysis (Sueyoshi & Goto, 2009; Delen et al., 2013; Manogna, 2022).

Researchers have proposed a plethora of data classification methodologies such as decision trees (Breiman et al., 1984), support vector machines (SVM) (Smola et al., 2004), neural networks (Zhang, 2000), ensemble methods (Breiman, 2001), Naïve Bayes (Zhang, 2004) and mathematical programming-based approaches (Xu & Papageorgiou, 2009). Concerning the last one, the incredible advancements witnessed in the last decades of both solvers for mixed integer optimisation and computer hardware have resulted in an astonishing increase in the computational capabilities of the mixed integer optimisation field, as shown in the literature (Bixby, 2012). These advancements have made it viable to develop various machine learning algorithms as mathematical programming-based methods suitable for small and medium-sized datasets. Exploiting this significant progress, a growing body of literature emphasises the use of mathematical programming-based classifiers due to their ability to achieve a balance between accuracy and interpretability.

Numerous classification applications necessitate interpretable models that can be easily comprehended by humans. In other words, people should be able to understand how the decision boundaries are formed between different classes and why certain labels are assigned to particular data points. Interpretable models play a crucial role in various domains as they serve as a bridge between domain experts and data scientists. When domain experts are faced with important decisions, incorporating data-driven insights can improve the quality of their outcomes, but this requires strong understanding of the model. The process of learning interpretable models is challenging, since interpretability and accuracy are often opposing objectives.

Interpretability in predictions is often achieved through the use of IF-THEN rules. Decision trees exemplify this concept and provide a balance between interpretability and accuracy. They are advantageous due to the fact that the splits are expressed in terms of the input features, eliminating the need for latent variables or representations, and they generate succinct, logical rules to make interpretable predictions. Furthermore, decision rule sets are classification rules in the context of DNF (Disjunctive Normal Form). In this framework, each conjunction is treated as a separate rule, and the rules themselves are not assigned any specific order. A positive prediction is generated when at least one of these rules is satisfied (Lakkaraju et al., 2016). Moreover, decision lists are a representation for Boolean functions, which can be easily learned from examples (Rivest, 1987). They differ from the decision rule sets because a decision list has an inherent order. For classifying a new instance, the rules are tried sequentially, and the predicted class is determined based on the first rule that covers the instance (Fürnkranz & Kliegr 2015). Lastly, associative classification is an interpretable technique that merges association rule mining and classification (Liu et al., 2000; Thabtah, 2007). During the rule discovery, only the class is examined as the consequent of a rule, instead of considering all attributes. Associative classification sifts through a vast number of rules, employing methods like pruning and ranking to isolate a subset of top-quality rules. Using this refined set, it constructs a classifier.

During the last decades, a plethora of mathematical programming formulations have addressed the classification problem with some of them trying to strike a balance between accuracy and interpretability. Initially, Gehrlein (1986) presented a mixed integer linear programming (MILP) formulation, which aims at the maximisation of correct classifications using a linear function for every class. Sueyoshi (2004) developed a two stage MILP formulation that incorporates data envelopment analysis for the weight estimation of a linear discriminant function by minimising the total deviation of misclassified instances. Busygin et al. (2007) proposed an optimisation-based approach, which can be used for feature selection, classification and outlier detection.

Mathematical programming has established as a valuable tool in defining a range of classification methods that were previously solved using heuristic techniques. Classification trees constitute a method that can be considered as one of the most powerful tools in statistics and machine learning (Hastie et al., 2008). They are widely used because they are rule-based and easy-to-interpret, when they are not very deep (Freitas, 2014). Popular heuristic methods, such as CART (Breiman et al., 1984), ID3 (Quinlan, 1986) and C4.5 (Quinlan, 1996) construct sub-optimal trees by greedily selecting a locally optimal split at each branch node, without considering the impact of future splits. As a result, the splits chosen at later nodes may negatively affect the generalisability of the tree. A potential approach to address this problem involves training the entire tree in a single-level process, allowing each split to be determined with complete awareness of all future splits.

Bertsimas and Dunn (2017) developed the Optimal Classification Trees (OCT) model, which formulates the classification tree learning process as an MILP problem. The OCT model determines the split rule at each branch node, class assignment to each leaf node, and the route of each sample from the root node to a leaf node. The objective function aims to minimise both the number of misclassified samples and the complexity of the tree, with the weight of the second term determined through tuning. Two versions of OCT are presented: one that uses orthogonal (univariate) splits at each branch node, and another that uses oblique (multivariate) splits at each branch node. Recently, many studies have used global exact optimisation methods to grow either univariate (Verwer & Zhang, 2017; Aghaei et al., 2019), or multivariate optimal classification trees of a pre-established depth (Blanquero et al. 2020;  Blanquero et al. 2021).

Another interpretable classification approach emerges through decision rule sets, which are usually found by widely used heuristic methods like RIPPER (Cohen, 1995) and CN2 (Clark, 1989). RIPPER uses a separate-and-conquer method to create simple rules while minimising overfitting. The dataset is divided for growing and pruning rules. Rules are grown by adding conditions until they perfectly classify the data, then pruned to reduce complexity. This process repeats until certain stopping criteria are met. An optimisation phase further improves the rule set, boosting accuracy and simplicity. Over the past few years, the problem of decision rule set learning is examined under modern optimisation lens. Lakkaraju et al. (2016) proposed a methodology that finds interpretable decision sets, examines different interpretability metrics and guarantees a near-optimal solution. Furthermore, the authors conducted a user study, which showed that it was easier for a participant to understand the structure of a decision set compared to a decision list. Dash et al. (2018) introduced a framework that formulates Boolean rule learning as an integer programming model, balancing accuracy and rule simplicity. Using column generation, a small subset of rules is generated and refined to reduce errors, while controlling complexity. New rules are added iteratively to improve predictive performance. This column generation framework makes the approach suitable for larger datasets. Moreover, Huynh et al. (2023) presented an efficient approach to predictive rule learning that uses greedy optimisation to learn locally optimal classification rules. Finally, Yu et al. (2021) addressed the issue of concise rule sets by encoding single-label rule learning problems as SAT programs and solving them using SAT solvers.

The power of mathematical programming has been demonstrated in support vector machines (SVM), as well (Carrizosa & Martin-Barragan, 2006); Liu et al. 2021). SVM training has been mathematically formulated as a convex quadratic problem with linear constraints, which can be solved to global optimality using non-linear solvers in the work presented by Carrizosa and Morales (2013). Blanco et al. (2020) proposed an SVM-based approach that maximises class separation, offering both MILP and MINLP formulations and a heuristic strategy involving dimensionality reduction and variable fixing. Generally, SVM is known to be sensitive to noisy data and outliers, so Blanco et al. (2022) proposed optimal SVM-based classifiers that account for label noise in the dataset. Different approaches were presented, including MILP and MINLP formulations that incorporate decisions on relabeling samples in the dataset. Finally, Bertsimas et al. (2021) formulated the sparse classification problem as a tractable binary convex optimisation problem for SVM.

Recently, many machine learning algorithms based on hyper-boxes have been introduced to tackle classification tasks (Alhroob et al., 2019; Khuat et al., 2021). Firstly, Simpson (1992) proposed a fuzzy min max neural network classifier. Fuzzy min max neural networks iteratively generate hyper-boxes, which are regions in an N-dimensional sample space defined by their minimum and maximum coordinates. Each hyper-box is linked to a fuzzy membership function that evaluates the degree to which an input sample belongs to a particular class. Fuzzy min max neural networks can generate explanation based on the rules deduced from the hyper-box min max values, but it cannot form a compact rule set, which is interpretable for end-users, because the number of hyper-boxes can be large (Khuat & Gabrys, 2020). Similar to hyper-box, the term “hyper-rectangle” has, also, been used to describe an N-dimensional sample space for classification tasks (Salzberg, 1991; Wettschereck, 1994; García et al. 2011; Martos et al. 2023).

Xu and Papageorgiou (2006) and Xu and Papageorgiou (2009) proposed an MILP model that adopts a hyper-box representation, which was inspired by previous work that addressed two-dimensional plant layout problems (Papageorgiou & Rotstein, 1998). By extending the model to N dimensions, hyper-boxes are formed to enclose a number of samples belonging to the respective class. These hyper-boxes are characterised by their centroid coordinate and the length along each dimension, effectively defining their boundaries. Constraints are included to prevent overlap between hyper-boxes of different classes. The objective of the model is to minimise the number of misclassified training samples. An iterative solution algorithm is presented that enables the addition of hyper-boxes in order to enclose a greater number of samples. Recently, an MILP formulation was presented by Liapis and Papageorgiou (2023). This work describes a single-level classification methodology, which recognises patterns using hyper-box representation. The hyper-boxes can be transformed into IF-THEN rules, but the number and the length of the generated rules are not restricted by the model.

Maskooki (2013) proposed a different iterative algorithm, which uses the bounds determined from previous iterations and eliminates the correctly classified samples from the training set in each iteration, instead of using the whole dataset for calculating new bounds. Kone and Karwan (2011) extended the data classification model and combined it with regression in order to predict the cost to serve new customers in the industrial gas business. Yang et al. (2015) introduced two new proposals to improve the performance of the model. The first improvement involves updating the sample weights during each iteration of the algorithm, which helps to prioritise the difficult samples in the subsequent iterations. The second improvement is a data space partition method that reduces the computational cost of the algorithm. Furthermore, a different formulation adopting a hyper-box representation was proposed by Üney and Türkay (2006), where boolean algebra was used to model the relationships among discrete variables. Finally, Kenger and Ozceylan (2023) integrated the improved online learning algorithm for general fuzzy min–max neural networks (IOL_GFMM) and the MILP model in order to improve the performance of the pure MILP model.

Concerning interpretability, some of the aforementioned methodologies generate IF-THEN rules (Bénard et al., 2021; Tan et al., 2020; Ooi et al., 2022). However, it is quite usual to have a large number of complex rules, which causes loss of interpretability. The goal of an interpretable classifier should be to construct a small number of short rules. An IF-THEN rule example is the following one:

$$\begin{aligned} {IF} \qquad \qquad {\left\{ \begin{array}{ll} (m1 \ge 0.7) \text { AND } \\ (m2 \le 0.2) \end{array}\right. }\qquad \qquad \qquad \qquad \qquad \implies \text { Class } c1 \end{aligned}$$

The rule states that if a sample value on attribute *m*1 is greater or equal to 0.7 and its value on attribute *m*2 is less or equal to 0.2, then the sample belongs to class *c*1. An attribute-value pair is called a literal and the number of literals is the length of the rule. Consequently, the length of the aforementioned rule is 2. It is obvious that short rules are easier to understand in comparison to long ones (Wang et al., 2015).

In this study, a single-level hyper-box approach for interpretable multi-class classification is proposed. This work extends the recently presented work by Liapis and Papageorgiou (2023), by improving the simplicity of its generated rules. This is achieved by incorporating the number of rules and their respective length in the mathematical model. In this way the hyper-box representation, which is not utilised by the existing rule-learning approaches, is employed for the extraction of rules. The objective of this paper is to demonstrate that hyper-box classification using mathematical programming leads to accurate and interpretable predictions.

Below, a summary of the contributions made in this paper is provided:

- The proposed approach tackles multi-class classification problems and derives a rule set using mathematical programming.
- The novel formulation uses a hyper-box representation and accounts for the number and the length of the generated rules. An extra term is added in the objective function, which minimises the complexity of the rule set.
- A tailored solution procedure is described, which contains i) a validation set approach to tune the weight of the complexity term in the objective function ii) the injection of a CART solution as a warm start in order to boost the performance of the solver.
- The power of mathematical programming is demonstrated through the integration of additional features in hyper-box classification problem, showcasing remarkable flexibility for the modeler. This adaptability makes the model extendable, enabling it to tackle a broad range of complex problems with different objectives, which in this case is the interpretability.
- A comprehensive computational comparison with five approaches is conducted by utilising 20 datasets. The objective of the comparison is to showcase the accuracy and interpretability of our approach. To assess the interpretability, the number and length of the rules generated by our method were compared to the other approaches.
- Our approach has a higher prediction accuracy on average of around 1.0–1.4% than *OCT*, 3.1–3.8% than *CART* and 6.3-7.3% than *WITT* at all examined levels of complexity. At the same time, it has a higher prediction accuracy on average of around 1.8–1.9% than *BRCG* and 0.8–1.4% than *JRIP* for the two highest examined levels of complexity. For the highest level of complexity examined, the number of rules produced by our approach is 0.24 lower than *OCT*, 1.85 lower than *CART*, 3.50 lower than *BRCG*, 3.98 lower than *WITT* and 0.56 higher than *JRIP*. Meanwhile, for the highest level of complexity examined, the average rule length of the proposed approach is 0.39 lower than *OCT*, 0.77 lower than *CART*, 0.20 lower than *BRCG*, 0.12 higher than *WITT* and 0.26 higher than *JRIP*.

The paper is structured as follows: Section 2, presents the proposed approach and explains the generation of IF-THEN rules. In Section 3, the implementation details are described and a number of benchmark classification datasets are employed to test the performance of our proposed method against other interpretable approaches. Finally, concluding remarks are drawn in Section 4.

## Methodology

### Problem statement

This section aims to introduce a novel classification methodology, namely *HOPE*. As mentioned earlier, the originally proposed MILP approach for hyper-box classification was presented by Xu and Papageorgiou (2009) and a single-level formulation was developed by Liapis and Papageorgiou (2023). However, in the previously proposed approaches it is possible that many hyper-boxes are generated in order to describe the patterns, which leads to the generation of many rules. Meanwhile, using many attributes to define the hyper-boxes leads to long and complex rules. A large number of long rules results in loss of interpretability. Therefore, an extension of the work presented by Liapis and Papageorgiou (2023) is proposed to generate a smaller number of accurate and simpler rules. Thus, the rationale of *HOPE* approach is to create a classifier that minimises the complexity of the rule set regarding the number and the length of the generated rules. The formulation includes constraints to ensure that the hyper-boxes enclose samples of the corresponding class, do not overlap with hyper-boxes from other classes, and satisfy interpretability requirements concerning the number of generated rules. In addition, the formulation is enhanced with symmetry breaking constraints and valid inequalities to improve computational efficiency. The objective function is a minimisation function and it consists of two terms; the training error and the summation of rule lengths.

Overall, the problem studied can be stated as follows:

*Given* : 

- Numerical values of *S* training samples with *M* attributes
- Classification of training samples into one of *C* classes
- Number of allowed hyper-boxes per class *I*
- Maximum number of active hyper-boxes $\Phi$

*Determine* : 

- Optimal bounds of hyper-boxes for every attribute

$$So \; as \; to:$$

- Minimise the training error and the summation of rule lengths

### Mathematical formulation

The indices, sets, parameters and variables associated with the model are presented below:

**Indices**

*s*: Sample $$(s=s_1,s_2,...,S)$$
*m*: Attribute$$(m =m_1,m_2,...,M)$$
*i*, *j*: Hyper-box $$(i,j=i_1,i_2,...,I)$$
*c*, *k*: Class $$(c,k=c_1,c_2,...,C)$$

**Sets**

$$C_{s}$$
Class which sample *s* belongs to
$$I_{c}$$
Set of hyper-boxes that belong to class *c*
$$S_{c}$$
Set of samples that belong to class *c*

**Parameters**

$$A_{sm}$$
Numeric value of sample *s* on attribute *m*
$$U_1,U_2, U_3$$
Suitably big numbers
$$\epsilon _m$$
Minimum distance between hyper-boxes that belong to different classes on attribute *m*
$$\Phi$$
User defined number that denotes the maximum number of active hyper-boxes
*W*: Weight coefficient

**Continuous variables**

$$UP_{cim}$$
Upper bound of hyper-box *i* of class *c* on attribute *m*
$$LO_{cim}$$
Lower bound of hyper-box *i* of class *c* on attribute *m*
$$\Lambda$$
Summation of rule lengths

**Binary variables**

$$E_{sci}$$
1, if sample *s* is correctly classified in hyper-box *i* of class *c*; 0 otherwise.
$$Y_{cikjm}$$
1, if hyper-box *i* of class *c* is on the left of hyper-box *j* of class *k* on attribute *m*; 0 otherwise.
$$NS_{ci}$$
1, if hyper-box *i* of class *c* is active; 0 otherwise.
$$Z_{cim}$$
1, if upper bound of hyper-box *i* of class *c* on attribute *m* is used to define the corresponding hyper-box; 0 otherwise.
$$O_{cim}$$
1, if lower bound of hyper-box *i* of class *c* on attribute *m* is used to define the corresponding hyper-box; 0 otherwise.

**Fig. 1:**
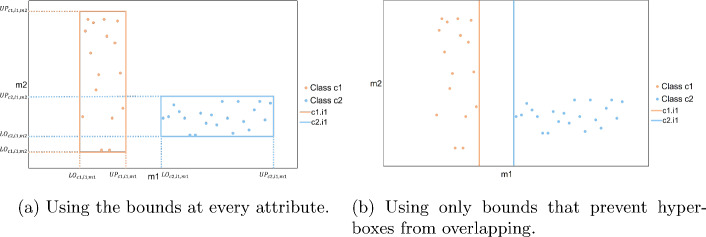
Hyper-boxes formed for a two dimensional dataset

In Figures 1a, 1b, a set of points is observed, where colour denotes two classes. This two dimensional dataset is used for illustrative purposes in order to demonstrate the creation of the hyper-boxes. In Figure 1a, all samples are enclosed inside a hyper-box of their corresponding class. The upper and lower bounds at every attribute are depicted. In Figure 1b, only the necessary bounds are depicted. The necessary bounds are those that prevent hyper-boxes from overlapping. In this case, the hyper-boxes do not overlap on attribute *m*1, because the hyper-box of class *c*1 is on the left of hyper-box of class *c*2. As it will be demonstrated later, by extracting IF-THEN rules from every hyper-box, the solution becomes more interpretable when a smaller number of bounds is kept. It is important to note that continuous data are assumed, which means that the upper and lower bounds of the hyper-boxes are modelled as continuous variables.

*Hyper-box enclosing constraints*

The first constraints are used to model whether a sample is enclosed in a hyper-box of its class. Constraints (1) state that if sample value $A_{sm}$ is higher than the lower bound of a hyper-box $LO_{cim}$ for every attribute *m*, then sample *s* is allowed to be included in hyper-box *i* of class *c*. Accordingly, constraints (2) allow a sample *s* to be included in a hyper-box *i* of class *c* if sample value $A_{sm}$ is lower than the upper bound of the hyper-box $UP_{cim}$ for every attribute *m*. This means that a sample can be enclosed in a hyper-box if its numerical value on every attribute is between the lower and upper bounds of the respective hyper-box. Meanwhile, if $E_{sci}=0$, the aforementioned constraints become redundant, since $U_{1}$ is big enough to satisfy the constraints. Constraints (3) allow every sample to be allocated to at most one hyper-box of its corresponding class, if correctly classified, thus avoiding possible double-counting.

1 $$\begin{aligned} A_{sm}\ge LO_{cim} - U_{1} \cdot (1- E_{sci}) \;\;\;\; \forall s,c \in C_{s},i \in I_{c} ,m\ \end{aligned}$$

2 $$\begin{aligned} A_{sm}\le UP_{cim} + U_{1} \cdot (1- E_{sci}) \;\;\;\; \forall s,c \in C_{s},i \in I_{c} ,m\ \end{aligned}$$

3 $$\begin{aligned} \sum _{i \in I_{c}} E_{sci}\le 1 \;\;\;\; \forall s,c \in C_{s} \end{aligned}$$

*Non-overlapping constraints*

Active hyper-boxes representing different classes are prohibited from overlapping, since they capture distinct patterns. Three sets of constraints are used to prevent overlapping. In constraints (4), if $Y_{cikjm}=1$, hyper-box *i* of class *c* precedes hyper-box *j* of class *k* on attribute *m*, because the upper bound of the first one , $UP_{cim}$, is lower than the lower bound of the second one, $LO_{kjm}$. In Figure 1b, it is shown that hyper-box *i*1 of class *c*1 has upper bound on attribute *m*1, which is lower than the lower bound of hyper-box *i*1 of class *c*2 on attribute *m*1. Subsequently, $Y_{c1,i1,c2,i1,m1}=1$ and these hyper-boxes do not overlap with each other on attribute *m*1. On the other hand, if two hyper-boxes overlap with each other on an attribute, then $Y_{cikjm}=0$ and the constraint becomes redundant.

The non-overlapping property must be ensured for all active hyper-boxes. In other words, if a hyper-box contains at least one sample, then it should not overlap with the hyper-boxes of different classes. The activation of a hyper-box is modelled by constraints (5), which enforce binary variable $NS_{ci}$ to be equal to 1 if hyper-box *i* of class *c* contains at least one sample. Subsequently, it is important to ensure that all active hyper-boxes do not overlap on at least one attribute. This is forced by constraints (6) for every combination of hyper-boxes that belong to different classes for at least one attribute. If hyper-box *i* of class *c* and hyper-box *j* of class *k* are both active, then it is required to satisfy that either $Y_{cikjm}=1$, or $Y_{kjcim}=1$ on at least one attribute. If at least one of the two hyper-boxes is not active, then constraint (6) becomes redundant.

4 $$\begin{aligned} & UP_{cim} +\varepsilon _{m} \le LO_{kjm}+ (U_{1}+\varepsilon _{m}) \cdot (1- Y_{cikjm}) \;\;\;\; \forall c,k \ne c,i \in I_{c} ,j \in I_{k},m\ \end{aligned}$$

5 $$\begin{aligned} & |S_{c}| \cdot NS_{ci} \ge \sum _{s \in S_{c}} E_{sci} \;\;\;\; \forall c,i\in I_{c} \end{aligned}$$

6 $$\begin{aligned} & \sum _{m}(Y_{cikjm}+Y_{kjcim}) \ge NS_{ci}+ NS_{kj} -1 \;\;\;\; \forall c,k<c, i\in I_{c}, j\in I_{k} \end{aligned}$$

Note that $\varepsilon _{m}$ is the minimum distance between hyper-boxes that belong to different classes to prevent them from sharing the same border. However, if $\varepsilon _{m}$ is too small, the MILP solver might face numerical instabilities. Hence, the goal is to find a value for $\varepsilon _{m}$ as high as possible that does not affect the feasibility of any valid solution. The biggest possible value is the smallest non-zero difference between two adjacent values on the examined attribute (Bertsimas & Dunn, 2017).

*Interpretability constraints*

Interpretability is a very important feature of a machine learning algorithm. In general, hyper-box models are easy to understand because they apply univariate splits and each hyper-box can be transformed into an IF-THEN rule. However, the number of generated IF-THEN rules can become large, which makes the set of rules difficult to follow. More specifically, the number of hyper-boxes that are used to enclose the samples of the dataset is equal to the number of rules that will be used to make the prediction for new samples. Consequently, the smaller the number of hyper-boxes that are generated the more interpretable the model is. Constraint (7) ensures that the number of active hyper-boxes is at most equal to $\Phi$, which is a user defined number.

7 $$\begin{aligned} \sum _{c} \sum _{i\in I_{c}} NS_{ci} \le \Phi \end{aligned}$$

Another feature that should be taken into consideration is the length of the rules. The bigger the length of a rule, the harder it is for the human brain to understand it. Subsequently, it is preferred to create short rules. The following constraints determine the summation of the lengths of all rules, which is equal to the summation of upper and lower bounds of attributes that are used to define the active hyper-boxes. More specifically, constraints (8) make the binary variable $Z_{cim}$ equal to 1 if there is at least one hyper-box *j* of class *k* that is on the right of hyper-box *i* of class *c* on attribute *m*. In this way, it is ensured that an upper bound for hyper-box *i* of class *c* should be determined on attribute *m*. Accordingly, constraints (9) make the binary variable $O_{cim}$ equal to 1 if there is at least one hyper-box *j* of class *k* that is on the left of hyper-box *i* of class *c* on attribute *m*. In this way, it is ensured that a lower bound for hyper-box *i* of class *c* should be determined on attribute *m*. From Figure 1b, as explained earlier it is true that $Y_{c1,i1,c2,i1,m1}=1$, which means that the upper bound of hyper-box *i*1 of class *c*1 on attribute *m*1 is used to define the corresponding hyper-box, so $Z_{c1,i1,m1}=1$. Accordingly, the lower bound of hyper-box *i*1 of class *c*2 on attribute *m*1 is used to define the corresponding hyper-box, so $O_{c2,i1,m1}=1$.

8 $$\begin{aligned} & \sum _{k\ne c } \sum _{j \in I_{k}} Y_{cikjm} \le U_{2} \cdot Z_{cim} \;\;\;\; \forall c,i \in I_{c},m \end{aligned}$$

9 $$\begin{aligned} & \sum _{k\ne c } \sum _{j \in I_{k}}Y_{kjcim} \le U_{2} \cdot O_{cim} \;\;\;\; \forall c,i \in I_{c},m \end{aligned}$$

Of course, the lower and upper bounds of the hyper-boxes should be considered in the cases that hyper-boxes are active, because only those hyper-boxes will be used to extract IF-THEN rules. Consequently, constraints (10) and (11) are added in order to enforce binary variables $Z_{cim}$ and $O_{cim}$ to be equal to 0 when a hyper-box *i* of class *c* is inactive. Constraint (12) states that the summation of upper and lower bounds of attributes, which are used to define all active hyper-boxes is at most equal to $\Lambda$.

10 $$\begin{aligned} & Z_{cim} \le NS_{ci} \;\;\;\; \forall c, i \in I_{c},m \end{aligned}$$

11 $$\begin{aligned} & O_{cim} \le NS_{ci} \;\;\;\; \forall c, i \in I_{c},m \end{aligned}$$

12 $$\begin{aligned} & \sum _{c} \sum _{i \in I_{c}} \sum _{m} (Z_{cim} + O_{cim}) \le \Lambda \end{aligned}$$

*Symmetry-breaking constraints*

Symmetry breaking constraints are added to avoid redundant equivalent solutions. More specifically, constraints (13) enforce the number of samples included in the lower indexed hyper-boxes to be higher than the number of samples included in the higher indexed hyper-boxes. In this way, some identical possible solutions are removed.

13 $$\begin{aligned} \sum _{s \in S_{c}} E_{s,c,i} \le \sum _{s \in S_{c}} E_{s,c,i-1} \;\;\;\; \forall c,i\in I_{c}, i \ge 2 \end{aligned}$$

*Valid inequalities*

To improve the computational performance, the mathematical formulation is strengthened using valid inequalities. Firstly, constraints (14) enforce the upper and lower bounds to become equal to zero if the corresponding hyper-box is not active. Afterwards, constraints (15) set binary variable $NS_{ci}$ equal to zero if no sample is correctly enclosed in hyper-box *i* of class *c*.

14 $$\begin{aligned} & UP_{cim} +LO_{cim} \le U_{3} \cdot NS_{ci} \;\;\;\; \forall c,i\in I_{c},m \end{aligned}$$

15 $$\begin{aligned} & NS_{ci} \le \sum _{s \in S_{c}}E_{sci} \;\;\;\; \forall c,i\in I_{c} \end{aligned}$$

*Objective function*

The objective function (16) contains two terms. The first term is the training error, which is equal to the number of misclassified samples divided by the total number of samples, multiplied by 100, so that it expresses a percentage. The second term expresses the summation of rule lengths. *W* is the weight coefficient of the second term, which allows one to find a trade-off between accuracy and interpretability.

16 $$\begin{aligned} min \;\; 100 \cdot \frac{\sum \limits _{s}( 1-\sum \limits _{ c \in C_{s}} \sum \limits _{i \in I_{c}}E_{sci} )}{|S|} + W \cdot \Lambda \end{aligned}$$

The overall optimisation problem is formulated as an MILP model, *HOPE* (**H**yper-box **OP**timisation-based l**E**arning), comprising constraints (1) - (16) and its goal is to minimise the training error and the summation of rule lengths.

### Testing phase

The arrangement of the hyper-boxes is completed during the training. During the testing phase, each testing sample is allocated to the hyper-box in which it is enclosed. However, it is possible that a sample is not enclosed in any of the hyper-boxes. In such cases, the allocation of the sample is based on the distance of the sample from all hyper-boxes (Xu & Papageorgiou, 2009; Maskooki, 2013; Yang et al., 2015; Liapis & Papageorgiou, 2023). The distance of testing sample *s* from hyper-box *i* of class *c* on attribute *m* is defined to be:

17 $$\begin{aligned} DIST_{scim} = max(0, A_{sm}-UP_{cim}, LO_{cim} - A_{sm}) \;\;\;\; \forall s, c , i\in I_{c}, m \end{aligned}$$

The total distance of sample s from hyper-box *i* of class *c* is given by:

18 $$\begin{aligned} DSI_{sci} = \sqrt{\sum _{m}DIST_{scim}^{2}} \;\;\;\; \forall s, c , i\in I_{c} \end{aligned}$$

Hence, having calculated the distances of testing samples from all hyper-boxes, the testing samples are allocated to their nearest derived hyper-box and assigned the membership of that hyper-box.

### Rule extraction

**Fig. 2:**
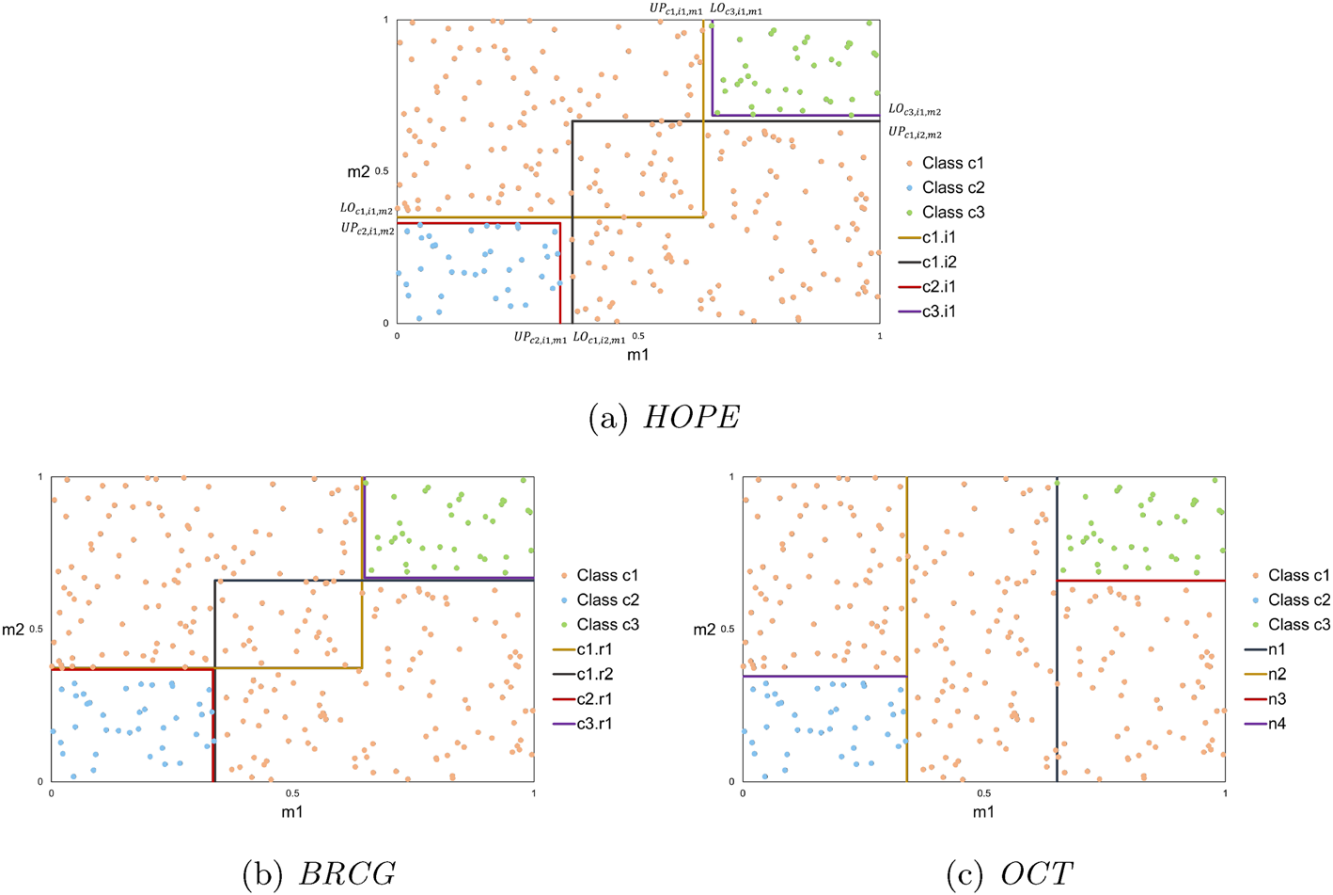
Data space partition of the approaches for the two dimensional dataset

In Figures 2a, 2b, 2c, another two dimensional dataset is used for illustrative purposes in order to demonstrate the rule extraction using different approaches. The dataset contains 300 samples and each one of them is assigned to one of the three classes.

Figure 2a visualises the hyper-boxes that are formed by *HOPE* model. It is noticed that all samples are correctly classified using 4 hyper-boxes. Also, the hyper-boxes of class *c*1 overlap with each other. The lower and upper bounds of the generated hyper-boxes are used to extract IF-THEN rules.

**Table 1:** Bounds of each hyper-box of *HOPE* solution

Hyper-box	Class	Attribute	Lower bound	Upper bound
i1	c1	m1	0	0.634
		m2	0.349	1
i2	c1	m1	0.363	1
		m2	0	0.666
i1	c2	m1	0	0.338
		m2	0	0.329
i1	c3	m1	0.653	1
		m2	0.685	1

Table 1 summarises the bounds of the hyper-boxes that are formed. Using these bounds, the corresponding IF-THEN rules can easily be generated. For example, hyper-box *i*1 of class *c*1 can be interpreted as the following rule:

$$\begin{aligned} \text {IF } (m1 \le 0.634) \text { AND } ( m2 \ge 0.349) \implies \text { Class } c1 \end{aligned}$$

Obviously, if the upper bound is 1 or the lower bound is 0, they can be omitted from the rule, because the data are normalised and will always satisfy these conditions. Therefore, the solution of *HOPE* for this dataset produces 4 IF-THEN rules and the length of every rule is equal to 2.

Similarly, the rules created by a rule-based methodology, namely Boolean decision rules via column generation (BRCG) (Dash et al., 2018) are depicted in Figure 2b. Naturally, *BRCG* is used for binary classification problems, but it can be extended to multi-class classification in the usual one-versus-rest manner. In this way, three binary classifiers (one for each class) are used for this dataset and all the generated rules are used to make the final predictions.

**Table 2:** Bounds of each rule of *BRCG* solution

Rule	Class	Attribute	Lower bound	Upper bound
r1	c1	m1	0	0.651
		m2	0.377	1
r2	c1	m1	0.335	1
		m2	0	0.668
r1	c2	m1	0	0.335
		m2	0	0.377
r1	c3	m1	0.651	1
		m2	0.668	1

Table 2 summarises the bounds of the rules that are generated. It is worth noting that some rules share boundaries, as a result some rules include the threshold ($\le , \ge$) and others do not ($<,>$), for example:

$$\begin{aligned} & \text {IF } (m1 \le 0.651) \text { AND } ( m2> 0.377) \implies \text { Class } c1\\ & \text {IF } (m1 > 0.651) \text { AND } ( m2 \ge 0.668) \implies \text { Class } c2 \end{aligned}$$

**Fig. 3:**
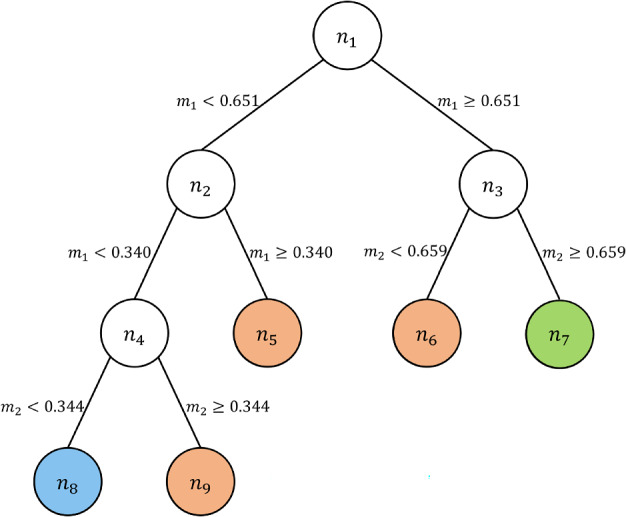
Tree structure for the two dimensional dataset

The same logic applies to the rule extraction from classification tree approaches. *OCT* (Bertsimas & Dunn, 2017) is applied to the two dimensional dataset and the structure of the resulting tree is shown in Figure 3. The tree contains 9 nodes, 4 of them apply splits (branch nodes) and 5 of them are leaf nodes. Leaf nodes $n_5, n_6,n_9$ represent class *c*1 and leaf nodes $n_7, n_8$ represent class *c*3 and *c*2, respectively.

In Table 3, the space that every leaf node represents is described. It is observed that the solution of *OCT* for this dataset produces 5 IF-THEN rules and the length of every rule is equal to 2.

**Table 3:** Bounds of each leaf node of *OCT* solution

Leaf node	Class	Attribute	Lower bound	Upper bound
n5	c1	m1	0.340	0.651
		m2	0	1
n6	c1	m1	0.651	1
		m2	0	0.659
n7	c3	m1	0.651	1
		m2	0.659	1
n8	c2	m1	0	0.340
		m2	0	0.344
n9	c1	m1	0	0.340
		m2	0.344	1

## Computational methodology

**Fig. 4:**
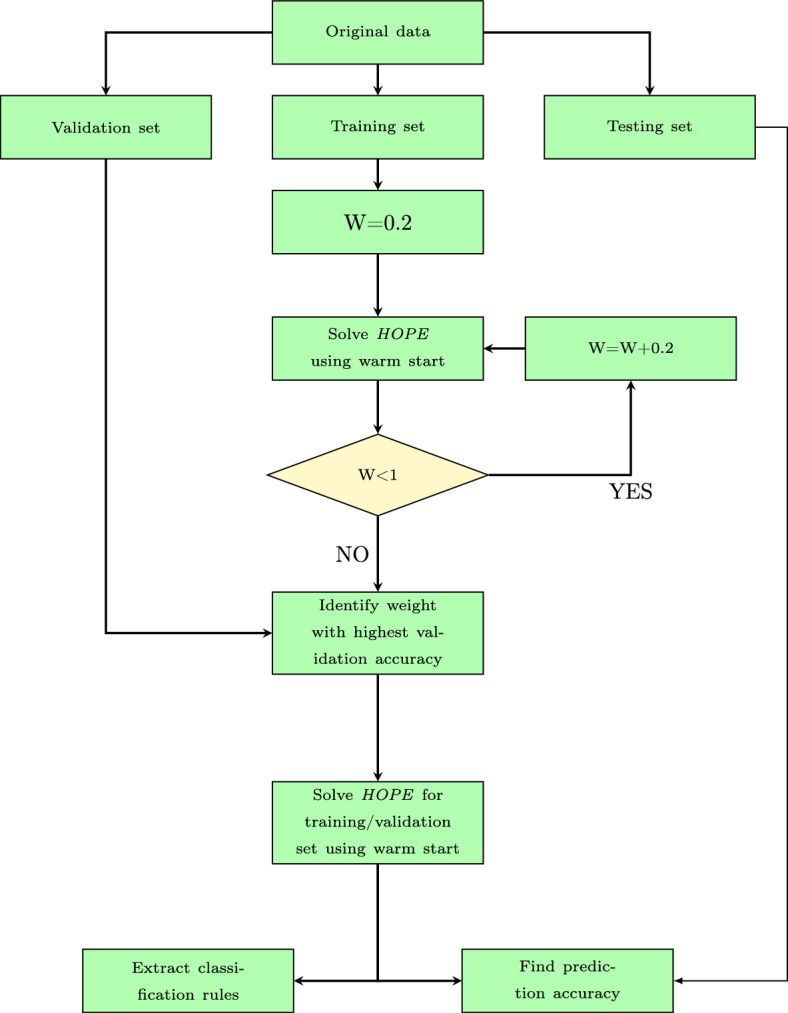
Implementation details of *HOPE* algorithm

In this section, the applicability of the proposed methodology is demonstrated by applying it to a number of datasets. Firstly, the implementation details of the full algorithm for training *HOPE* classifier are described, as shown in Figure 4. Firstly, the dataset is divided into training/validation set (70 %) and testing set (30 %). The training/validation set is further divided into training set (70 %) and validation set (30 %). Afterwards, the feature values of the dataset are normalised. The scaler was first fitted without using the testing set in order to avoid data leakage. Then, the scaler parameters were applied to transform the testing set. The validation set is used to effectively tune the value of the hyperparameter of this model. More specifically, the value of the complexity parameter *W* is determined by validation set approach (Hastie et al., 2008) and the grid used in the experiments is $\{W1,W2,W3,W4,W5\}= \{0.2,0.4,0.6,0.8,1\}$. All validation runs have a time limit equal to 100 s and a *CART* solution is injected as a warm start before starting the solver in order to boost its performance. Thus, the weight with the highest validation accuracy is selected for the final *HOPE* model, which also benefits from a warm start solution of *CART*. The time limit is set equal to 1800 s. Results about the impact of warm start and the objective function value improvement across time can be found in Appendix A. Finally, the prediction accuracy is calculated and the corresponding IF-THEN rules are extracted. The proposed methodology is applied to a number of datasets shown in Table 4, and it is compared to other literature approaches. All datasets can be downloaded from UCI machine learning repository (Dua & Graff, 2017) and are widely used as benchmarks to compare the performance of different classification methods. It is important to note that if the dataset contains categorical features they are converted using one-hot encoding. The implementation of the proposed algorithm was conducted in GAMS (General Algebraic Modeling System) (GAMS Development Corporation, 2017) and the selected solver was GUROBI (Gurobi Optimization, LLC, 2023).

**Table 4:** Datasets

Dataset	Abbreviation	Samples	Attributes	Classes
Iris	I	150	4	3
Teaching Assistant Evaluation	TAE	151	54	3
Wine	W	178	13	3
Parkinsons	P	195	22	2
Seeds	S	210	7	3
Image Segmentation	IS	210	19	7
Glass	G	214	9	6
Thyroid New	TN	215	5	3
Heart Disease Cleveland	HDC	297	13	5
E-coli	E	336	7	8
Ionosphere	ION	351	34	2
Dermatology	DER	358	34	6
Data User Modelling	DUM	403	5	4
Breast Cancer Wisconsin	BCW	569	30	2
Balance	B	625	4	3
Pima Indian Diabetes	PID	768	8	2
Connectionist	CNN	990	10	11
Banknote Authentication	BA	1372	4	2
Contraceptive	CNTR	1473	9	3
Wi-fi	WF	2000	7	4

The proposed methodology *HOPE* is compared with two univariate classification tree approaches; *CART* (Breiman et al., 1984) and *OCT* (Bertsimas & Dunn, 2017) and three rule-based approaches; Wittgenstein implementation (abbreviated as *WITT*) of RIPPER algorithm (Moscovitz, 2020), WEKA implementation of RIPPER algorithm, called *JRIP* (Frank et al., 2005) and *BRCG* (Dash et al., 2018; Arya et al., 2019). *CART* and *OCT*, were implemented using Scikit-learn library (Pedregosa et al., 2011) and InterpretableAI library (Interpretable AI, LLC, 2023), respectively.

Varying levels of rule complexity lead to different predictions and corresponding prediction accuracies. Therefore, we examine three versions of each classifier, each reflecting a different level of rule complexity. In this way, it is possible to observe how accuracy changes as the complexity increases, in terms of the number of rules and rule length. Balancing complexity and accuracy is crucial, as simpler models with fewer, shorter rules are generally easier to interpret, but may trade off some accuracy. Conversely, more complex models can increase accuracy at the cost of rule simplicity. Depending on the application, different levels of complexity may be acceptable, hence some fields prioritise interpretability, while others may allow for more complexity to maximise accuracy. To explore this trade-off for the examined methodologies, different parameter grids are used to control the levels of rule complexity. Meanwhile, for a baseline comparison, the out-of-the-box performance of the examined classifiers is provided in Appendix B.

As explained earlier, classification trees produce solutions that can be interpreted as IF-THEN rules. For classification tree approaches, the maximum number of rules is equal to the maximum number of leaf nodes, which is equal to $2^D$, where *D* is the depth of the tree. Subsequently, a tree, whose depth is equal to $D=2$, has up to 4 leaf nodes and up to 4 IF-THEN rules can be generated. The same logic applies to the rest of the depths. As far as *HOPE* is concerned, the maximum number of rules is equal to the user defined number $\Phi$. In order to ensure a fair comparison with the classification tree, $\Phi$ is set equal to the maximum number of leaf nodes of the examined depth. Thus, *HOPE* algorithm for $\Phi =4$ is expressed as *HOPE-4* and allows the generation of the same number of rules to *OCT* of depth $D=2$, which is expressed as *OCT-2*. Meanwhile, the number of allowed hyper-boxes per class is set $I=\frac{\Phi }{|C|}$, rounding up when needed. The comparison of the methodologies will be conducted for three levels of complexity. The different levels of complexity are expressed by the maximum allowed rules, which are equal to 4, 8, 16. So, for the first level of complexity of the classification trees a grid of $D=\{1,2\}$ is examined, while for the second and third level of complexity, grids of $D=\{1,2,3\}$ and $D=\{1,2,3,4\}$ are, respectively, considered. While, *HOPE* is examined for maximum number of hyper-boxes $\Phi =4,8,16$ at the respective level of complexity.

As far as the comparison with the rule-based approaches is concerned, *WITT* and *BRCG* are naturally binary classifiers, so a one-versus-rest strategy is employed and the total number of generated rules cannot be inherently restricted. Unlike *CART*, *OCT*, and *HOPE*, none of the three rule-based approaches allows for limiting the total number of rules with an upper bound. Therefore, some key parameters related to rule complexity will be restricted to maintain comparability. Regarding *WITT*, three levels of complexity (*WITT-1*, *WITT-2*, *WITT-3*) are accommodated by limiting the maximum number of rules per class ($\mu$) to the following grids: $\{1,2\}$, $\{1,2,3\}$, $\{1,2,3,4\}$. Concerning *BRCG*, parameter $\lambda$ is used to balance the trade-off between accuracy and simplicity of the generated rules. Thus, three levels of complexity, named *BRCG-1*, *BRCG-2*, *BRCG-3*, are examined by searching the following grids for $\lambda$: $\{0.01,0.05,0.1\}$, $\{0.005,0.01,0.05,0.1\}$, $\{0.001,0.005,0.01,0.05,0.1\}$. In the cae of *JRIP*, parameter *min weight* is used to accommodate three levels of rule complexity. More specifically, *JRIP-1*, *JRIP-2*, *JRIP-3* correspond to the following grids: $\{8,10\}$, $\{4,6,8,10\}$, $\{0,2,4,6,8,10\}$. Finally, it is important to note that all the approaches compared receive the same training and testing subsets at every iteration.

Here, the results of all approaches are reported in an attempt to compare them in terms of accuracy and rule simplicity. Table 5 presents a comparison of the average prediction accuracy with the associated standard deviation per dataset of the compared approaches for the first level of complexity. More specifically, the presented experiments include *CART* and *OCT* for depth $D=\{1,2\}$, *WITT* for maximum number of rules per class $\mu$: $\{1,2\}$, *BRCG* for $\lambda$: $\{0.01,0.05,0.1\}$, *JRIP* for $min \; weight$: $\{8,10\}$ and *HOPE* for maximum number of hyper-boxes $\Phi =4$. Similarly, in Table 6 the second level of complexity is examined, including *CART* and *OCT* for depth $D=\{1,2,3\}$, *WITT* for maximum number of rules per class $\mu$: $\{1,2,3\}$, *BRCG* for $\lambda$: $\{0.005,0.01,0.05,0.1\}$, *JRIP* for $min \; weight$: $\{4,6,8,10\}$ and *HOPE* for maximum number of hyper-boxes $\Phi =8$. Likewise, the results for the third level of complexity are presented in Table 7, which comprises of *CART* and *OCT* for depth $D=\{1,2,3,4\}$, *WITT* for maximum number of rules per class $\mu$: $\{1,2,3,4\}$, *BRCG* for $\lambda$: $\{0.001,0.005,0.01,0.05,0.1\}$, *JRIP* for $min \; weight$: $\{0,2,4,6,8,10\}$ and *HOPE* for maximum number of hyper-boxes $\Phi =16$. Furthermore, Appendix C provides results concerning the average number of the extracted rules and their average length for every dataset using the aforementioned methodologies at the different levels of complexity. Moreover, in Appendix D, violin plots are used to describe the distribution of the prediction accuracy of every methodology for several datasets. Finally, the statistical significance when comparing all the pairs of classifiers at each level of complexity can be found in Appendix E.

**Fig. 5:**
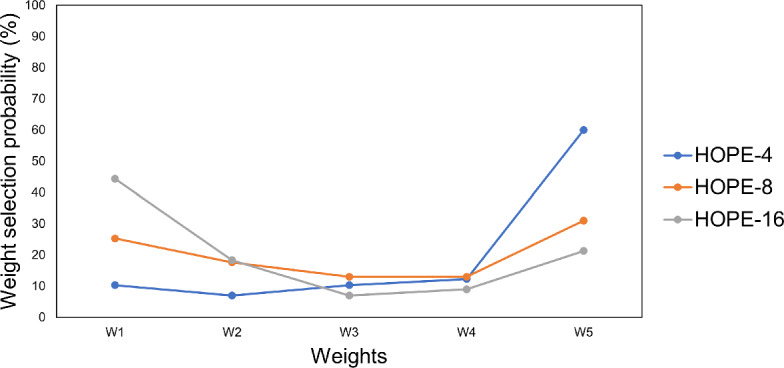
Weight selection probability (%) after validation step across all datasets

Figure 5 illustrates the frequency that a candidate weight of those in the grid is selected across all datasets in *HOPE-4*, *HOPE-8*, *HOPE-16*. As explained earlier, the weight with the highest validation accuracy is selected for the final *HOPE* model. In case two weights have the same validation accuracy, then the highest weight is used for the final *HOPE* model. It is noteworthy that all weights are selected a considerable amount of times during the validation stage. At the same time, *W*5 is the most frequently selected for *HOPE-4* and *HOPE-8* and *W*1 for *HOPE-16*. Of course, the user can experiment with different values in the grid or even different validation procedures in order to determine the balance between the two terms in the objective function.

**Table 5:** Average prediction accuracy (%) of *CART*, *OCT*, *WITT*, *BRCG*, *JRIP* and *HOPE* for the first level of complexity per dataset

Dataset	CART-2	OCT-2	WITT-1	BRCG-1	JRIP-1	HOPE-4
I	94.22±3.61	94.52±3.43	78.96±8.15	92.00±3.70	92.44±5.61	94.22±3.61
TAE	33.48±3.79	35.07±7.44	33.77±5.61	36.23±5.82	29.28±4.48	36.38±5.29
W	87.65±5.13	88.02±6.12	74.20±8.43	90.62±6.21	89.01±5.39	94.96±3.50
P	82.15±3.60	83.62±3.20	75.71±3.48	85.65±3.91	84.29±3.42	85.99±4.13
S	89.10±2.65	89.42±2.70	67.72±7.02	87.09±5.43	84.23±6.34	90.26±3.32
IS	39.37±8.64	49.63±4.36	72.28±7.46	75.13±5.52	74.39±7.24	47.83±4.98
G	60.82±6.52	61.33±6.12	54.26±6.42	56.41±8.73	58.77±7.18	62.97±6.50
TN	91.28±2.79	92.41±3.62	92.00±2.88	93.33±4.12	93.13±2.74	93.85±2.51
HDC	54.52±4.05	53.41±4.05	55.04±5.71	49.11±4.18	53.26±4.11	52.30±3.62
E	77.03±4.03	78.28±5.21	69.57±4.42	78.28±4.39	77.49±4.32	78.02±2.99
ION	90.19±1.82	88.81±5.08	85.35±3.93	89.12±2.55	90.75±2.65	90.63±2.35
DER	64.57±3.60	75.93±3.43	85.37±2.87	92.22±2.34	91.54±3.10	77.78±4.01
DUM	84.74±4.24	85.90±2.95	73.77±4.33	89.42±2.56	88.48±2.69	85.62±3.07
BCW	92.01±2.47	91.23±2.80	81.68±2.77	93.45±2.91	92.90±2.18	93.22±1.48
B	66.28±3.46	66.70±1.72	66.99±2.93	73.01±4.21	74.61±2.71	66.99±1.97
PID	73.74±3.05	74.05±3.13	72.67±2.80	74.78±3.00	74.52±1.98	74.29±2.80
CNN	24.22±1.57	29.65±2.59	35.11±3.57	28.24±2.39	57.80±3.08	30.53±2.03
BA	90.45±1.23	91.02±0.94	72.41±2.43	95.84±0.46	97.07±0.95	92.22±0.91
CNTR	47.74±1.96	53.71±2.22	46.05±2.16	52.46±1.64	53.41±2.40	55.48±1.59
WF	90.01±9.60	95.74±0.62	80.51±2.07	92.50±1.32	96.76±0.65	95.80±0.58
*Average*	*71.68*	*73.92*	*68.67*	*76.25*	*77.71*	*74.95*

**Table 6:** Average prediction accuracy (%) of *CART*, *OCT*, *WITT*, *BRCG*, *JRIP* and *HOPE* for the second level of complexity per dataset

Dataset	CART-3	OCT-3	WITT-2	BRCG-2	JRIP-2	HOPE-8
I	94.52±3.52	94.37±3.23	87.41±6.22	93.19±3.58	92.00±4.79	94.37±3.62
TAE	35.94±5.72	40.00±8.58	35.51±7.17	40.58±5.25	32.61±6.35	39.42±7.18
W	91.73±3.50	91.36±5.88	84.69±4.56	91.85±5.73	88.02±5.10	94.81±3.65
P	84.41±4.38	87.34±4.71	75.71±3.48	85.65±3.86	84.52±3.44	87.80±4.64
S	89.31±2.75	90.05±3.09	82.33±4.98	87.30±5.34	86.67±6.80	90.69±3.37
IS	57.04±7.76	74.07±8.35	74.07±5.46	77.88±6.06	75.24±7.88	84.87±4.29
G	65.13±6.22	64.10±5.08	58.15±7.71	59.28±8.41	62.77±6.34	64.41±5.44
TN	92.41±2.72	93.95±2.83	94.56±2.86	92.92±3.72	93.13±2.97	94.26±2.14
HDC	54.37±3.71	53.48±3.91	53.93±4.09	48.59±4.28	53.33±3.76	54.07±4.71
E	80.73±4.02	80.53±5.25	73.33±3.70	78.09±4.34	80.40±3.41	81.85±4.00
ION	89.69±3.37	87.48±4.50	87.67±2.72	89.56±3.40	88.93±2.47	90.82±2.54
DER	78.52±3.89	88.02±2.84	89.01±2.89	92.28±2.28	92.22±2.57	94.20±2.80
DUM	91.18±2.15	91.63±2.59	81.49±6.97	89.97±2.30	91.35±2.02	91.02±2.78
BCW	92.94±1.55	93.33±2.01	88.97±2.17	94.62±1.17	93.33±1.93	93.14±1.42
B	67.94±2.13	68.97±2.67	71.63±2.96	76.06±2.96	75.46±2.62	70.57±2.50
PID	72.81±3.78	74.05±3.13	72.64±2.77	74.92±2.86	74.69±2.81	74.49±2.42
CNN	35.87±1.96	42.45±3.38	40.04±3.79	40.02±4.37	64.98±3.32	45.01±2.23
BA	92.96±1.06	96.13±0.89	77.86±1.42	96.86±0.87	97.30±0.54	95.76±1.61
CNTR	51.19±1.99	56.08±1.30	44.60±2.66	52.56±1.70	52.52±3.28	53.88±2.93
WF	96.58±0.94	96.90±0.47	88.59±3.67	93.18±0.81	97.23±0.43	96.46±0.53
*Average*	*75.76*	*78.22*	*73.11*	*77.77*	78.84	*79.59*

**Table 7:** Average prediction accuracy (%) of *CART*, *OCT*, *WITT*, *BRCG*, *JRIP* and *HOPE* for the third level of complexity per dataset

Dataset	CART-4	OCT-4	WITT-3	BRCG-3	JRIP-3	HOPE-16
I	94.22±3.79	94.81±3.41	88.44±5.27	93.63±3.02	91.70±5.59	94.22±3.42
TAE	38.55±6.32	43.19±8.24	34.78±5.99	43.77±7.77	32.90±6.59	42.32±9.75
W	92.10±3.91	90.86±5.76	84.57±6.30	91.60±5.65	88.02±4.53	94.81±3.59
P	83.28±4.28	87.34±4.71	75.71±3.48	85.54±4.10	84.86±2.73	89.15±4.19
S	88.78±2.56	90.26±3.32	83.28±5.37	87.41±5.78	89.10±5.08	91.01±3.51
IS	68.89±9.52	84.55±5.75	72.59±5.10	77.67±6.04	77.35±6.80	86.35±4.81
G	64.72±6.66	68.51±5.38	54.56±8.77	57.23±8.05	65.74±5.93	68.62±7.12
TN	92.21±2.89	93.74±2.28	93.33±2.90	92.92±3.68	94.05±2.63	94.56±1.36
HDC	54.30±3.85	53.70±3.62	54.59±3.11	48.52±4.33	53.19±3.39	52.67±3.56
E	79.93±3.03	80.20±5.42	71.35±4.58	77.82±4.01	81.19±3.39	83.04±3.89
ION	89.50±2.03	87.61±4.53	89.43±2.46	90.88±2.30	89.87±2.27	91.95±2.03
DER	90.25±2.76	91.85±2.27	90.62±3.72	92.04±3.13	92.41±2.85	93.89±2.88
DUM	91.18±1.64	91.40±2.66	83.91±5.15	89.97±2.30	91.52±2.08	90.74±3.14
BCW	92.83±2.09	93.76±1.50	88.97±2.44	94.04±1.09	93.02±2.01	91.70±2.63
B	67.80±2.25	70.64±3.44	73.62±4.36	77.84±3.25	74.86±3.12	74.33±3.16
PID	73.25±2.98	74.05±3.13	72.90±2.46	74.92±2.86	75.82±2.69	74.72±2.93
CNN	46.71±1.75	51.36±2.33	40.47±2.97	59.44±2.23	66.64±3.44	54.66±3.31
BA	94.90±1.10	97.94±1.23	82.43±2.87	97.59±0.52	97.96±0.58	97.33±1.12
CNTR	55.17±1.73	56.06±1.27	45.23±2.37	52.37±1.82	52.28±2.64	55.48±1.78
WF	97.26±0.68	97.16±0.53	91.59±2.42	94.99±0.75	96.80±0.45	96.48±0.54
*Average*	*77.79*	*79.95*	*73.62*	*79.01*	*79.46*	*80.90*

**Fig. 6:**
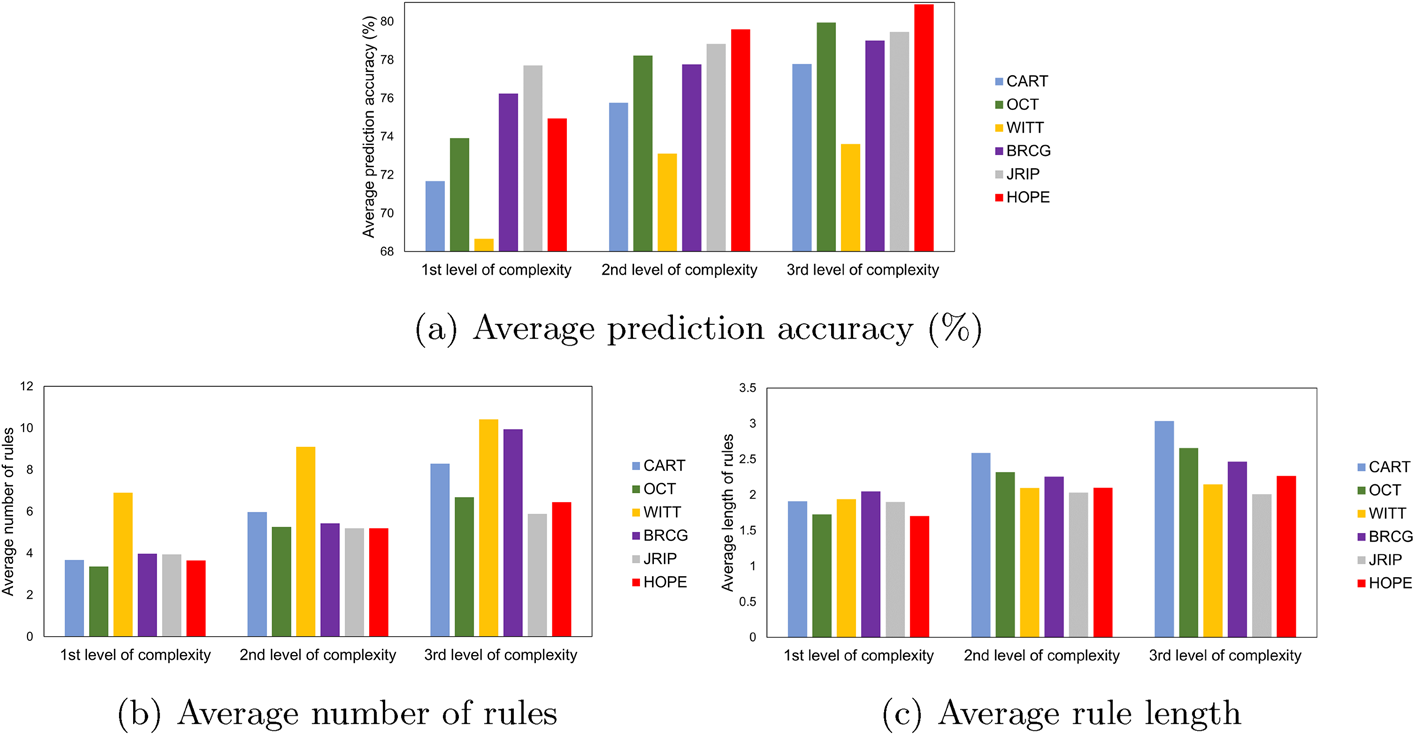
Accuracy and rule set simplicity comparison for three levels of complexity

Figure 6a depicts the average prediction accuracy of all the approaches across all datasets for three levels of complexity. Concerning the first level of complexity, *JRIP-1* shows the best predictive performance and *BRCG-1* comes second, followed by *HOPE-4*, *OCT-2*, *CART-2*, *WITT-1*. Here, it is worth noting that *BRCG* and *JRIP* do not provide the feature of restricting the total number of generated rules. As a result they much better in some datasets with a high number of classes like Image Segmentation, in which the average prediction accuracy of *BRCG-1* and *JRIP-1* are 75.13 % and 74.39 %, respectively, while the average prediction accuracy of *HOPE-4* is 48.57 %. Thus, the absence of restrictions on the total number of generated rules significantly boosts their average performance. Moving to the second and third levels of complexity, it becomes evident that *HOPE* consistently outperforms the other approaches, followed by *JRIP* and *OCT*, respectively, with *BRCG*, *CART* and *WITT* ranking lower.

In terms of rule set simplicity, Figures 6b, 6c illustrate the average number of rules and the average rule length across all datasets for three levels of complexity. Of course, the methodologies that produce the least amount of rules and with the shortest lengths are sought after. At the first level of complexity, *OCT-2* produces the fewest rules on average, followed by *HOPE-4*, *CART-2*, *JRIP-1*, *BRCG-1* and *WITT-1*. Concerning the second level of complexity, *HOPE-8* exhibits the lowest average rule number and it is succeeded by *JRIP-2*,*OCT-3*, *BRCG-2*, *CART-3* and *WITT-2*. Lastly, *JRIP-3* *HOPE-16*, *OCT-4* produce fewest rules on average for the highest level of complexity examined, followed by *CART-4*, *BRCG-3* and *WITT-3*. As far as the average rule length is concerned, *HOPE-4* produces the shortest rules on average for the first level of complexity, followed by *JRIP-1*, *OCT-2*, *CART-2*, *WITT-1* and *BRCG-1*. Finally, for the second and third level of complexity, *JRIP*, *WITT* and then *HOPE* emerge as the most efficient in terms of generating the shortest rules on average, succeeded by *BRCG*, *OCT* and *CART*.

**Fig. 7:**
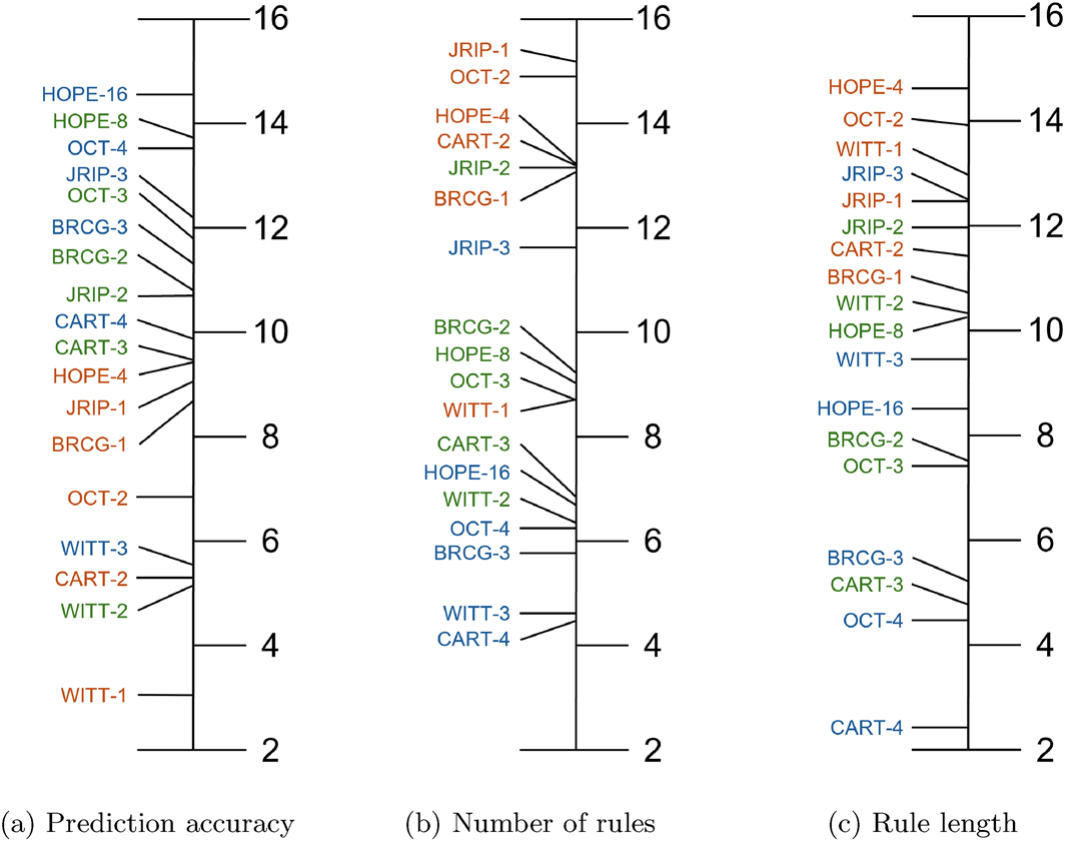
Rankings of predictive performance and rule set simplicity of the methods

Apart from averaging classification accuracies and rule simplicity metrics across different datasets, generating rankings based on algorithm performance on related datasets provides complementary insights for evaluating machine learning models. Thus, a scoring strategy is utilised in order to assess the relative competitiveness of the examined approaches. In this strategy, each approach is assigned a score based on its performance. The best performer receives 18 points, while the worst performer is awarded 1 point. The final ranking is determined by calculating the average score obtained by each method across all datasets. Meanwhile, different colours are used to distinguish between different levels of complexity: orange represents the first level, green the second level, and blue the third level. In Figure 7a, the prediction accuracy ranking is depicted and it is observed that *HOPE-16* has the best overall score. Concerning *HOPE-8*, it outperforms all other approaches of same and even higher complexity. Furthermore, *HOPE-4* is superior to the other approaches of the first level of complexity, including *BRCG-1* and *JRIP-1*, which appeared better in the previous comparison of averaging the accuracies across all datasets in Figure 6a, because of their performance at specific datasets.

In Figures 7b and 7c, the top-performing approaches are the ones that create the least amount of rules and the shortest ones, respectively. The ranking that accounts for the number of rules, which is depicted in Figure 7b, ranks *OCT-2* as the methodology that creates the fewest rules, followed by *JRIP-1*, *HOPE-4*, *CART-2*, and *BRCG-1*. Regarding the second and third level of complexity, *HOPE-8* and *HOPE-16* outscore all their respective counterparts, except for *JRIP*. Lastly, Figure 7c illustrates the ranking of the approaches concerning the average rule length. *HOPE-4* exceeds all other approaches, while both *HOPE-8* and *HOPE-16* outperform the other approaches of their corresponding level of complexity except for *WITT* and *JRIP*. The presented rankings indicate that the method consistently exhibits high predictive accuracy and surpasses other methods in the majority of cases. Meanwhile, in terms of rule simplicity, the model seems to be competitive and sometimes even superior compared to other approaches.

## Concluding remarks

In this work, a single-level hyper-box classifier is proposed, which addresses the topic of multi-class classification and interpretability. A single-level approach (*HOPE*) has been proposed, which adopts a hyper-box representation and is formulated mathematically as a Mixed Integer Linear Programming (MILP) model. Key decisions of *HOPE* involve optimal sizing and arrangement of hyper-boxes of each class in order to identify dataset patterns. The hyper-boxes enclose as many samples of the corresponding class as possible. At the same time, they are not allowed to overlap with each other if they represent different classes. As far as the interpretability is concerned, it is restricted by controlling the maximum number of the generated rules and it is enhanced by inserting the summation of rule lengths in the objective function. Furthermore, the interpretability of the model is highlighted by the extraction of IF-THEN rules from hyper-boxes. Finally, the effectiveness of the proposed methodology has been demonstrated through twenty literature datasets. Based on the computational results, our approach appears to outperform other alternatives in terms of predicting outcomes. Concerning the number of generated rules and their respective length, our model appears to be competitive with, and sometimes even better than other approaches. This highlights the suitability of our approach for real-world datasets when the goal is to achieve high prediction accuracy, while also maintaining interpretability.

## Figures and Tables

**Fig. 8:**
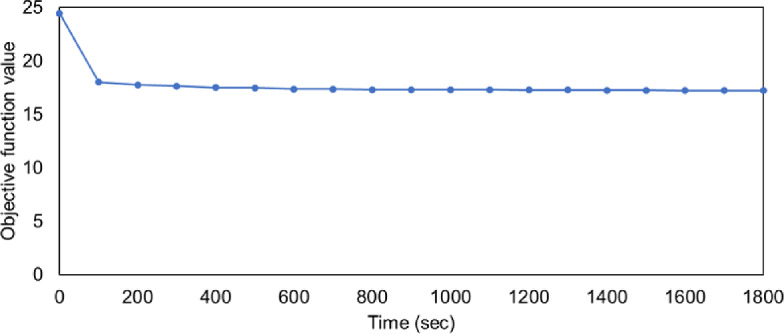
Average objective function value across time

**Fig. 9:**
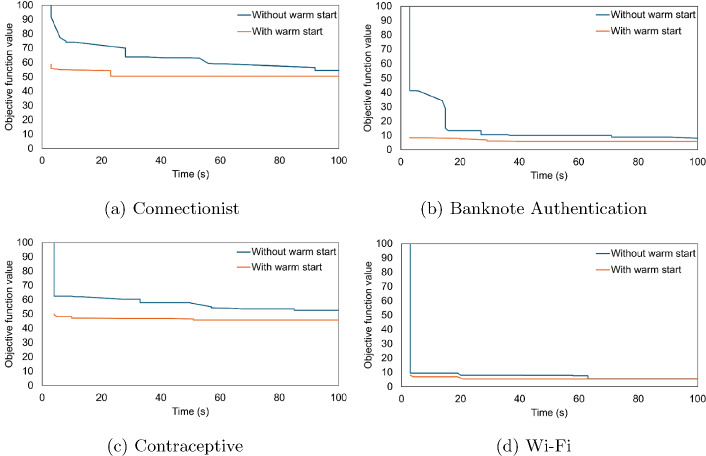
Objective function value across time with and without warm start injection

**Fig. 10:**
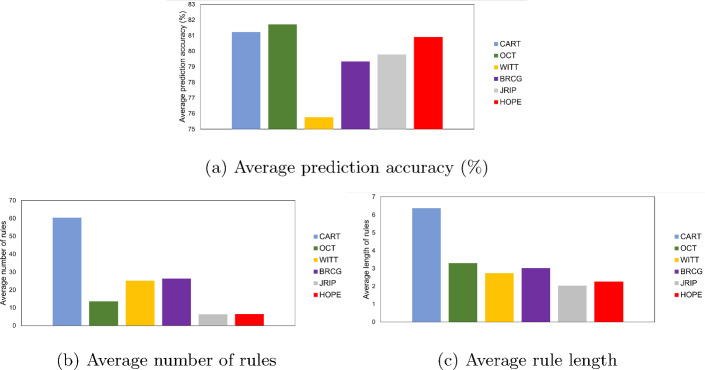
Accuracy and rule set simplicity of out-of-the-box *CART*, *OCT*, *WITT*, *BRCG*, *JRIP* and *HOPE-16*

**Fig. 11:**
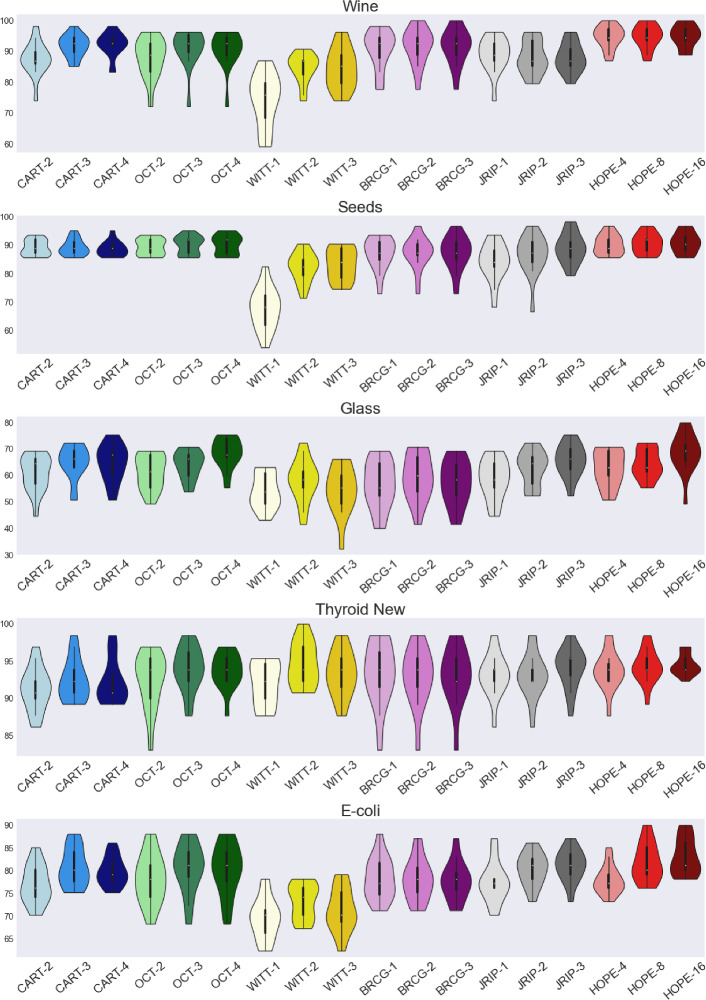
Prediction accuracy distribution of the examined approaches for three levels of complexity for the Wine, Seeds, Glass, Thyroid New, E-coli

**Fig. 12:**
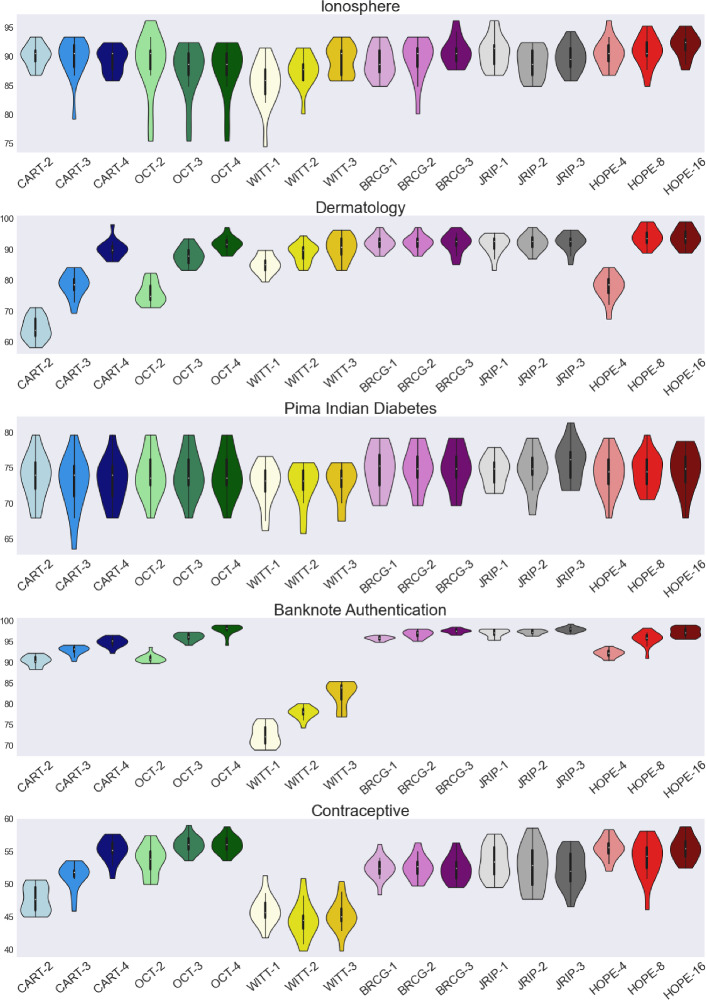
Prediction accuracy distribution of the examined approaches for three levels of complexity for Ionosphere, Dermatology, Pima Indian Diabetes, Banknote Authentication, Contraceptive

## Data Availability

The benchmark datasets are available online (Data Source available in manuscript).

## Appendix A: Computational results

To evaluate candidate time-limits for the validation runs, experiments were conducted across all 20 datasets and three complexity levels (HOPE-4, HOPE-8, HOPE-16). Figure 8 illustrates the average improvement in the objective function over time for all the runs. The objective function value at time equal to 0 is the CART solution, which is injected as a warm start. Notably, the objective function shows significant improvement within the first 100 s, in contrast to negligible improvement in the remaining time. In this way, a time limit of 100 s is selected for the validation runs in order to secure a good quality solution within a reasonable timeframe.

To evaluate the impact of using a warm start, additional runs were conducted comparing the computational performance with and without the warm start injection. Figure 9 depict the solutions found by *HOPE-16* for the four largest datasets (Connectionist, Banknote Authentication, Contraceptive, and Wi-Fi) with and without warm start. The results demonstrate that using CART as a warm start significantly improves the MILP solution within the 100-second time frame. More specifically, the objective function value when using warm start is constantly lower than the objective function value without using warm start across the entire 100-s range. Finally, it is shown that CART solution is improved significantly within 100 s.

## Appendix B: Comparison with out-of-the-box approaches

The approaches used for comparison with the proposed methodology were previously constrained by rule set complexity to accommodate the different levels of complexity. It is interesting to compare their out-of-the-box performance with *HOPE*, as this provides a baseline comparison. Tables 8, 9, 10 and Fig. 10 present the average accuracy, number of rules, and rule length of the classifiers for the examined datasets. For reference, the results for the highest complexity level of the proposed method, *HOPE-16*, are included, although it is constrained to 16 total rules. Our proposed approach is only outperformed by *CART* and *OCT* in terms of accuracy, a difference largely attributable to the Connectionist dataset, where both CART and OCT are allowed to produce more rules and better capture underlying patterns. However, these methods generate a significantly larger number of longer rules, which reduces interpretability. More specifically, *CART* produces nearly 10 times the number of rules that *HOPE-16* produces, while *OCT* produces more than twice as many. Moreover, the average rule length of *HOPE-16* is 1.03 lower than *OCT* and 4.1 lower than *CART*. In this way, it is shown that both *CART* and *OCT* sacrifice rule simplicity to some extent in order to improve prediction accuracy. Regarding the comparison with *WITT*, *BRCG* and *JRIP*, it is observed that *HOPE-16* clearly outperforms them in terms of prediction accuracy and, in most cases, is also superior in rule simplicity.

**Table 8:** Average prediction accuracy of out-of-the-box *CART*, *OCT*, *WITT*, *BRCG*, *JRIP* and *HOPE-16* per dataset

Dataset	CART	OCT	WITT	BRCG	JRIP	HOPE-16
I	95.11	94.81	89.93	93.19	93.33	94.22
TAE	56.38	45.65	35.94	51.59	32.61	42.32
W	91.85	90.86	89.51	93.09	89.63	94.81
P	85.20	87.34	73.79	84.75	85.99	89.15
S	90.37	90.26	85.71	88.99	89.31	91.01
IS	87.51	86.77	71.75	78.73	76.83	86.35
G	67.18	66.15	59.08	58.67	67.18	68.62
TN	93.23	93.33	93.54	93.33	94.67	94.56
HDC	48.22	54.22	52.74	44.22	54.07	52.67
E	77.76	81.65	69.17	78.35	80.73	83.04
ION	88.74	87.99	89.43	89.69	88.49	91.95
DER	94.14	94.07	91.05	92.10	91.79	93.89
DUM	90.36	91.40	84.08	87.33	91.85	90.74
BCW	92.44	93.80	90.96	93.96	93.41	91.70
B	74.68	75.89	77.34	76.84	76.88	74.33
PID	69.58	74.14	71.37	72.15	74.60	74.72
CNN	78.14	74.88	54.28	66.49	66.53	54.66
BA	98.20	98.33	95.89	98.09	97.67	97.33
CNTR	48.07	55.38	45.10	49.61	53.30	55.48
WF	97.37	97.34	94.77	95.50	96.96	96.48
*Average*	*81.23*	*81.72*	*75.77*	*79.33*	*79.79*	*80.90*

**Table 9:** Average number of rules of out-of-the-box *CART*, *OCT*, *WITT*, *BRCG*, *JRIP* and *HOPE-16* per dataset

Dataset	CART	OCT	WITT	BRCG	JRIP	HOPE-16
I	7.60	3.27	12.07	8.67	2.40	3.07
TAE	47.73	21.27	6.87	37.80	2.67	7.07
W	7.27	4.13	16.27	7.00	2.67	4.20
P	11.73	4.33	7.60	4.87	2.47	6.00
S	11.60	4.07	15.47	12.13	3.27	4.40
IS	16.13	10.33	20.53	18.53	7.87	8.33
G	35.20	14.33	18.67	26.27	6.13	9.67
TN	10.93	6.47	15.40	10.13	3.40	4.67
HDC	66.33	5.40	14.47	39.87	2.53	7.80
E	41.27	9.47	31.67	27.93	7.73	5.93
ION	18.60	4.33	6.07	8.87	3.93	6.40
DER	14.60	7.80	19.87	13.87	6.67	6.67
DUM	22.47	7.40	33.93	21.87	6.73	6.33
BCW	15.87	5.93	9.60	5.80	2.93	3.93
B	113.93	14.93	20.00	78.67	8.33	7.20
PID	95.93	3.27	5.73	23.93	2.53	4.67
CNN	120.33	113.40	124.60	99.80	34.67	14.07
BA	23.67	10.07	31.53	7.07	6.07	8.40
CNTR	484.13	9.87	11.60	48.27	3.73	5.20
WF	41.93	11.80	81.27	25.13	9.40	4.93
*Average*	*60.36*	*13.59*	*25.16*	*26.32*	*6.31*	*6.45*

**Table 10:** Average rule length of out-of-the-box *CART*, *OCT*, *WITT*, *BRCG*, *JRIP* and *HOPE-16* per dataset

Dataset	CART	OCT	WITT	BRCG	JRIP	HOPE-16
I	3.55	1.82	1.98	2.40	1.43	1.36
TAE	10.08	5.06	1.51	2.02	1.16	2.37
W	3.03	2.12	1.88	2.09	1.63	2.07
P	3.85	1.93	1.78	2.86	1.97	2.21
S	3.97	2.02	2.31	2.55	1.62	1.86
IS	6.09	3.91	2.68	2.60	1.66	2.81
G	6.51	3.87	3.00	3.27	2.24	3.20
TN	4.27	2.88	1.96	2.67	1.46	1.87
HDC	6.95	2.54	3.05	3.62	2.39	2.70
E	6.36	3.26	2.99	3.23	1.85	2.55
ION	5.77	2.33	2.03	2.09	1.41	1.74
DER	6.04	3.86	1.84	2.68	1.93	3.62
DUM	5.45	2.95	3.24	3.20	1.66	2.11
BCW	4.52	2.60	2.82	2.70	1.97	1.51
B	8.76	4.80	3.13	3.83	2.98	2.50
PID	8.34	1.63	2.88	4.29	2.29	1.60
CNN	8.84	7.85	4.20	3.70	2.83	3.23
BA	5.15	3.60	3.86	2.54	2.29	1.98
CNTR	12.59	3.29	3.54	4.78	3.29	2.04
WF	7.36	3.58	4.10	3.27	2.64	1.98
*Average*	*6.37*	*3.30*	*2.74*	*3.02*	*2.03*	*2.27*

## Appendix C: Average number of rules and average rule lengths

See Tables 11, 12, 13, 14, 15 and 16.

**Table 11:** Average number of rules of *CART*, *OCT*, *WITT*, *BRCG*, *JRIP* and *HOPE* for the first level of complexity per dataset

Dataset	CART-2	OCT-2	WITT-1	BRCG-1	JRIP-1	HOPE-4
I	3.00	3.00	6.00	3.10	2.07	3.07
TAE	2.93	2.87	3.40	4.53	0.73	3.80
W	3.87	3.53	6.00	4.07	2.00	4.00
P	2.40	2.27	2.00	2.13	1.20	3.47
S	3.93	3.27	5.93	3.94	2.20	3.87
IS	3.27	4.00	13.40	8.47	5.93	4.00
G	4.00	3.87	9.33	6.87	2.33	4.00
TN	3.60	3.47	5.60	3.90	2.00	3.67
HDC	3.73	1.80	7.07	1.73	0.33	2.73
E	4.00	3.73	12.60	4.80	3.87	4.00
ION	3.73	2.93	1.93	3.07	2.13	3.00
DER	3.93	4.00	11.87	6.00	5.20	4.00
DUM	4.00	4.00	8.00	6.33	4.33	4.00
BCW	3.73	2.73	2.00	1.73	2.00	3.40
B	3.87	3.20	5.00	4.33	4.87	3.20
PID	3.60	2.87	1.53	2.13	2.73	2.93
CNN	4.00	4.00	21.93	4.20	21.73	4.00
BA	4.00	4.00	2.00	2.00	4.33	4.00
CNTR	4.00	3.80	4.47	2.20	3.40	4.00
WF	4.00	4.00	8.00	4.07	5.60	4.00
*Average*	*3.68*	*3.37*	*6.90*	*3.98*	*3.95*	*3.66*

**Table 12:** Average number of rules of *CART*, *OCT*, *WITT*, *BRCG*, *JRIP* and *HOPE* for the second level of complexity per dataset

Dataset	CART-3	OCT-3	WITT-2	BRCG-2	JRIP-2	HOPE-8
I	3.53	3.20	8.47	4.00	2.20	3.07
TAE	6.13	4.93	3.80	10.73	2.20	6.00
W	6.00	4.20	8.80	4.80	2.07	4.13
P	4.47	4.33	2.00	2.73	1.87	5.07
S	4.87	3.87	8.87	4.87	2.60	4.27
IS	4.47	6.40	15.33	10.33	6.93	7.60
G	6.93	7.17	11.73	9.73	4.93	6.67
TN	5.07	5.27	7.53	5.29	2.00	4.93
HDC	5.40	3.13	6.67	2.67	1.27	4.27
E	8.00	6.10	15.87	6.20	5.43	5.20
ION	4.07	3.53	2.93	4.77	2.60	4.33
DER	5.00	6.40	16.07	6.53	5.47	6.67
DUM	7.73	6.93	11.33	6.87	5.13	6.53
BCW	6.07	4.00	3.00	2.07	2.73	3.87
B	8.00	5.40	7.00	6.07	6.67	5.13
PID	4.13	2.87	1.93	2.16	2.40	3.67
CNN	7.73	8.00	31.07	8.27	30.93	8.00
BA	8.00	6.60	3.00	2.73	5.20	5.53
CNTR	6.53	5.87	4.87	2.67	4.13	3.87
WF	7.40	7.13	11.87	5.17	7.20	5.07
*Average*	*5.98*	*5.27*	*9.11*	*5.43*	*5.20*	*5.19*

**Table 13:** Average number of rules of *CART*, *OCT*, *WITT*, *BRCG*, *JRIP* and *HOPE* for the third level of complexity per dataset

Dataset	CART-4	OCT-4	WITT-3	BRCG-3	JRIP-3	HOPE-16
I	4.20	3.27	9.80	6.33	2.13	3.07
TAE	9.07	8.47	4.07	21.67	2.53	7.07
W	6.67	4.13	11.33	5.47	2.40	4.20
P	4.47	4.33	2.00	3.20	2.13	6.00
S	6.27	4.07	11.33	7.60	3.27	4.40
IS	5.93	8.33	16.00	11.53	7.47	8.33
G	9.20	10.40	12.13	16.53	5.80	9.67
TN	6.00	6.13	7.73	6.33	2.87	4.67
HDC	5.40	3.73	7.20	4.87	2.20	7.80
E	12.07	6.60	16.80	9.20	6.93	5.93
ION	5.07	3.60	3.67	6.07	2.53	6.40
DER	6.07	7.40	17.00	9.07	6.40	6.67
DUM	10.27	7.40	14.20	6.87	6.00	6.33
BCW	7.67	5.07	3.80	3.07	2.93	3.93
B	14.13	8.40	8.73	17.00	7.60	7.20
PID	4.93	2.87	2.07	2.16	2.67	4.67
CNN	13.40	15.53	37.33	46.27	33.47	14.07
BA	12.20	8.27	4.00	3.80	6.07	8.40
CNTR	10.93	5.67	4.80	3.80	3.80	5.20
WF	11.93	10.13	14.40	8.00	8.60	4.93
*Average*	*8.29*	*6.69*	*10.42*	*9.94*	*5.89*	*6.45*

**Table 14:** Average rule length of *CART*, *OCT*, *WITT*, *BRCG*, *JRIP* and *HOPE* for the first level of complexity per dataset

Dataset	CART-2	OCT-2	WITT-1	BRCG-1	JRIP-1	textbfHOPE-4
I	1.67	1.67	1.70	1.56	1.38	1.36
TAE	1.86	1.44	1.40	2.19	0.83	1.76
W	1.96	1.84	1.42	1.82	1.87	2.03
P	1.20	1.16	1.80	1.98	1.60	1.61
S	1.98	1.76	1.72	1.80	1.57	1.76
IS	1.76	2.00	1.93	2.24	1.50	1.83
G	2.00	1.96	2.48	2.78	1.60	2.05
TN	1.87	1.82	1.51	2.23	1.53	1.66
HDC	1.87	0.76	2.85	1.87	0.93	1.46
E	2.00	1.91	1.87	2.33	1.81	2.03
ION	1.91	1.62	1.37	1.18	1.07	1.33
DER	1.98	2.00	1.84	1.94	1.83	2.25
DUM	2.00	2.00	1.83	2.23	1.83	1.50
BCW	1.87	1.42	1.50	1.60	1.87	1.52
B	1.93	1.73	3.04	2.82	3.11	1.42
PID	1.80	1.47	1.50	1.73	2.38	1.26
CNN	2.00	2.00	3.30	2.35	3.15	1.82
BA	2.00	2.00	1.33	2.50	2.39	1.68
CNTR	2.00	1.93	2.73	1.56	3.22	2.00
WF	2.00	2.00	1.68	2.27	2.58	1.75
*Average*	*1.89*	*1.72*	*1.94*	*2.05*	*1.90*	*1.70*

**Table 15:** Average rule length of *CART*, *OCT*, *WITT*, *BRCG*, *JRIP* and *HOPE* for the second level of complexity per dataset

Dataset	CART-3	OCT-3	WITT-2	BRCG-2	JRIP-2	HOPE-8
I	1.92	1.78	1.81	2.19	1.44	1.36
TAE	2.91	2.18	1.54	2.27	1.31	2.25
W	2.63	2.14	1.51	1.81	1.68	2.06
P	1.95	1.93	1.93	2.22	1.92	1.91
S	2.27	1.96	1.73	2.18	1.57	1.81
IS	2.36	2.75	2.18	2.49	1.64	2.72
G	2.83	2.67	2.58	3.04	2.10	2.87
TN	2.44	2.45	1.67	2.29	1.50	1.95
HDC	2.32	1.51	2.79	2.07	2.28	1.89
E	3.00	2.55	2.08	2.44	1.95	2.47
ION	2.03	1.87	1.37	1.41	1.21	1.52
DER	2.41	2.68	1.84	2.09	1.79	3.64
DUM	2.96	2.84	2.07	2.28	1.64	2.16
BCW	2.55	2.03	1.67	2.10	1.87	1.60
B	3.00	2.55	3.43	3.07	3.06	2.01
PID	1.93	1.47	1.53	1.83	2.34	1.48
CNN	2.96	3.00	3.43	3.08	2.95	2.71
BA	3.00	2.56	1.62	2.13	2.38	1.80
CNTR	2.75	2.61	3.02	1.97	3.48	1.78
WF	2.88	2.87	2.18	2.17	2.57	2.02
*Average*	*2.56*	*2.32*	*2.10*	*2.26*	*2.03*	*2.10*

**Table 16:** Average rule length of *CART*, *OCT*, *WITT*, *BRCG*, *JRIP* and *HOPE* for the third level of complexity per dataset

Dataset	CART-4	OCT-4	WITT-3	BRCG-3	JRIP-3	HOPE-16
I	2.17	1.82	1.85	2.02	1.29	1.36
TAE	3.87	3.02	1.41	2.21	1.21	2.37
W	2.83	2.12	1.64	1.87	1.61	2.07
P	1.81	1.93	1.87	2.46	1.86	2.21
S	2.64	2.02	1.89	2.21	1.47	1.86
IS	2.99	3.30	2.28	2.45	1.61	2.81
G	3.30	3.32	2.60	3.14	2.19	3.20
TN	2.78	2.76	1.74	2.34	1.45	1.87
HDC	2.32	1.83	2.78	2.54	2.51	2.70
E	3.57	2.70	2.16	2.59	1.97	2.55
ION	2.33	1.91	1.51	1.70	1.13	1.74
DER	2.86	3.24	1.82	2.44	1.82	3.62
DUM	3.40	2.95	2.28	2.26	1.66	2.11
BCW	2.89	2.37	1.80	2.33	1.89	1.51
B	3.78	3.25	3.14	3.53	2.95	2.50
PID	2.07	1.47	1.63	1.83	2.23	1.60
CNN	3.82	3.97	3.45	3.76	2.85	3.23
BA	3.69	3.23	1.77	2.61	2.30	1.98
CNTR	3.62	2.57	2.87	2.32	3.55	2.04
WF	3.62	3.36	2.46	2.74	2.63	1.98
*Average*	*3.02*	*2.66*	*2.15*	*2.47*	*2.01*	*2.27*

## Appendix D: Prediction accuracy distributions

The violin plot outlines illustrate kernel probability density, i.e. the width of the shaded area reflects the data proportion within that range. Meanwhile, the median is represented by a white dot, and the interquartile range is indicated by a thick black bar at the center. Figures 11, 12 show that *HOPE* manages to perform on-par with the other methodologies. Furthermore, it is worth noting that comparing *HOPE-4*, *HOPE-8* and *HOPE-16*, the results illustrate that by increasing the maximum number of rules, the prediction accuracy distribution for most datasets improves significantly.

## Appendix E: Statistical significance

The pairwise statistical significance of the prediction accuracy of the classifiers is examined using t-test for paired samples. T-test is commonly employed to assess if the difference between the performance of two classifiers over various data sets is random (Virtanen et al., 2020). The p-values, which are presented in Table 17, indicate the statistical significance between the prediction accuracy of *HOPE* and the rest of the classifiers for all three examined levels of complexity. It is shown most p-values are less than 0.05, which makes the differences statistically significant.

**Table 17:** Pairwise p-value indicating the statistical significance between the prediction accuracy of the examined classifiers

	WITT-1	BRCG-1	JRIP-1	CART-2	OCT-2
HOPE-4	0.020	0.456	0.212	0.001	0.015

	WITT-2	BRCG-2	JRIP-2	CART-3	OCT-3
HOPE-8	$5.59 \cdot 10^{-5}$	0.011	0.568	0.021	0.046

	WITT-3	BRCG-3	JRIP-3	CART-4	OCT-4
HOPE-16	$6.40 \cdot 10^{-6}$	0.039	0.167	0.004	0.039

## Appendix F: Samples outside all hyper-boxes

The testing samples that fall outside all hyper-boxes are assigned to the nearest hyper-box, as explained in Section 2.3. Table 18 presents the percentage of samples that lie outside of all hyper-boxes. It is observed that the number of such samples increases with complexity, which is attributed to the fragmentation of the space as the number of hyper-boxes increases. Table 19 presents the average prediction accuracy of samples that are outside of all hyper-boxes. In some datasets, like Iris, Parkinsons, Breast Cancer Wisconsin and Pima Indian Diabetes, assigning samples to the nearest hyper-box based on distance results in high prediction accuracy. However, in other datasets, like Balance, Contraceptive and Wi-fi, the number of samples outside all hyper-boxes is either 0 (making the average prediction accuracy not applicable) or very small, leading to inconclusive results.

**Table 18:** Average percentage of samples that are outside of all hyper-boxes per dataset

Dataset	HOPE-4	HOPE-8	HOPE-16
I	5.93	5.93	5.78
TAE	3.48	3.62	5.94
W	17.16	17.28	17.53
P	17.29	18.98	20.68
S	9.63	10.48	11.11
IS	18.62	16.30	17.78
G	13.13	15.69	20.82
TN	5.33	5.44	5.95
HDC	5.04	5.70	7.85
E	6.07	6.53	7.13
ION	0.50	0.75	1.70
DER	0.74	1.17	1.05
DUM	1.49	3.42	3.47
BCW	7.72	7.80	8.03
B	0.00	0.04	0.07
PID	1.93	2.11	2.14
CNN	11.18	9.52	12.62
BA	0.91	0.95	1.28
CNTR	0.05	0.05	0.05
WF	0.00	0.00	0.00
*Average*	*6.31*	*6.93*	*7.95*

**Table 19:** Average prediction accuracy of samples that are outside of all hyper-boxes per dataset

Dataset	HOPE-4	HOPE-8	HOPE-16
I	95.00	95.00	94.87
TAE	50.00	44.44	28.26
W	82.73	88.57	87.32
P	90.20	86.90	85.25
S	79.12	82.83	81.90
IS	24.43	70.78	69.05
G	42.19	49.67	57.14
TN	78.85	84.91	86.21
HDC	54.41	49.35	35.85
E	54.35	64.65	62.96
ION	12.50	58.33	51.85
DER	66.67	57.89	52.94
DUM	70.37	70.97	63.49
BCW	91.25	90.50	84.47
B	N/A	0.00	0.00
PID	88.06	83.56	83.78
CNN	16.06	32.55	36.65
BA	75.00	83.05	78.48
CNTR	0.00	0.00	0.00
WF	N/A	N/A	N/A

## Code availability

Not available online.
